# Characterization of Gut Microbiota Compositions along the Intestinal Tract in *CD163/pAPN* Double Knockout Piglets and Their Potential Roles in Iron Absorption

**DOI:** 10.1128/spectrum.01906-22

**Published:** 2023-01-10

**Authors:** Xiu-Ling Zhang, Yan-Rong Zhou, Song-Song Xu, Si Xu, Yu-Jian Xiong, Kui Xu, Chang-Jiang Xu, Jing-Jing Che, Lei Huang, Zhi-Guo Liu, Bing-Yuan Wang, Yu-Lian Mu, Shao-Bo Xiao, Kui Li

**Affiliations:** a State Key Laboratory of Animal Nutrition, Institute of Animal Sciences, Chinese Academy of Agricultural Sciences, Beijing, People’s Republic of China; b College of Animal Science and Technology, Nanjing Agricultural University, Nanjing, People’s Republic of China; c State Key Laboratory of Agricultural Microbiology and Key Laboratory of Preventive Veterinary Medicine in Hubei Province, College of Veterinary Medicine, Huazhong Agricultural University, Wuhan, People’s Republic of China; d Shenzhen Branch, Guangdong Laboratory of Lingnan Modern Agriculture, Genome Analysis Laboratory of the Ministry of Agriculture and Rural Affairs, Agricultural Genomics Institute at Shenzhen, Chinese Academy of Agricultural Sciences, Shenzhen, People’s Republic of China; Chengdu University

**Keywords:** 16S rRNA, iron absorption, gut microbiota, piglet, carboxylic acids

## Abstract

The gut microbiota is known to play a role in regulating host metabolism, yet the mechanisms underlying this regulation are not well elucidated. Our study aimed to characterize the differences in gut microbiota compositions and their roles in iron absorption between wild-type (WT) and *CD163*/*pAPN* double-gene-knockout (DKO) weaned piglets. A total of 58 samples along the entire digestive tract were analyzed for microbial community using 16S rRNA gene sequencing. The colonic microbiota and their metabolites were determined by metagenomic sequencing and untargeted liquid chromatography-mass spectrometry (LC-MS), respectively. Our results showed that no alterations in microbial community structure and composition were observed between DKO and WT weaned piglets, with the exception of colonic microbiota. Interestingly, the DKO piglets had selectively increased the relative abundance of the *Leeia* genus belonging to the Neisseriaceae family and decreased the *Ruminococcaceae_UCG_014* genus abundance. Functional capacity analysis showed that organic acid metabolism was enriched in the colon in DKO piglets. In addition, the DKO piglets showed increased iron levels in important tissues compared with WT piglets without any pathological changes. Pearson’s correlation coefficient indicated that the specific bacteria such as *Leeia* and *Ruminococcaceae_UCG_014* genus played a key role in host iron absorption. Moreover, the iron levels had significantly (*P < *0.05) positive correlation with microbial metabolites, particularly carboxylic acids and their derivatives, which might increase iron absorption by preventing iron precipitation. Overall, this study reveals an interaction between colonic microbiota and host metabolism and has potential significance for alleviating piglet iron deficiency.

**IMPORTANCE** Iron deficiency is a major risk factor for iron deficiency anemia, which is among the most common nutritional disorders in piglets. However, it remains unclear how the gut microbiota interacts with host iron absorption. The current report provides the first insight into iron absorption-microbiome connection in *CD163*/*pAPN* double knockout piglets. The present results showed that carboxylic acids and their derivatives contributed to the absorption of nonheme iron by preventing ferric iron precipitation.

## INTRODUCTION

Gut microbiota colonizing the animal digestive tract, is a key factor in regulating host energy harvest and metabolism (1). Shotgun metagenome sequencing of pig gut microbiome identified ~7.7 million nonredundant genes that were annotated to bacteria, viruses, eukaryotes, and archaea (2). In recent years, many studies have shown that the composition of dominant gut microbes can significantly affect average daily weight gain, body weight, and feed efficiency in pigs (3, 4). However, we still have little understanding of the relationship between gut microbiota and these production traits in pigs.

The gut microbiota plays an important role in host iron absorption (5, 6). Numerous studies have shown that the microbiota may influence iron absorption within the gastrointestinal tracts (GIT) (7). In general, iron is primarily absorbed by duodenal enterocytes; the remainder enters the colonic lumen, where it is available to the gut microbiota (8). Previous studies suggested that gut microbiota such as *Lactobacillus*, *Enterococcus,* and Enterobacteriaceae family members was associated with iron deficiency (9, 10). When pH is high, end products of microbial fermentation such as tartaric, malic, and citric acids are thought to prevent the precipitation of ferric iron, enhancing iron absorption (11). However, it remains unclear how the gut microbiota interacts with host iron absorption.

Iron is a central trace element, particularly essential for the growth and development of animals and necessary for erythropoiesis (12, 13). Iron deficiency is one of the most common nutritional disorders in piglets. Neonatal piglets are susceptible to iron deficiency anemia mainly due to special physiological conditions, such as low levels of iron stores at birth, low iron concentration in porcine milk, heightened growth trajectories after birth, and immature iron absorption pathways (14, 15). Supplementation of inorganic iron in piglets plays an important role in preventing iron deficiency anemia (16). Due to the lack of regulations associated with iron elements contained in feed ingredients, excessive iron supplementation can occur, which may cause intestinal inflammation and diarrhea in piglets (17, 18). The health problems caused by both iron deficiency and iron overload have seriously hindered the development of pig-raising industries.

The double-gene-knockout (DKO) model has both *CD163* and *pAPN* knocked out (via CRISPR/Cas9) and is resistant to the infection with both porcine reproductive and respiratory syndrome virus (PRRSV) and transmissible gastroenteritis virus (TGEV) (19). In this study, we compared the microbial community diversity and composition in different intestinal segments (duodenum, jejunum, ileum, cecum, and colon) and feces between DKO and WT weaned piglets through 16S rRNA sequencing. Then, we detected the enriched colonic microbial function and metabolites in the DKO piglets by implementing metagenomic sequencing and untargeted metabolomics, respectively. Moreover, this study utilized the DKO Large White piglet as a model to study the associations between gut microbiota and iron absorption. This study presents a panel of gut microbial species significantly associated with differences in iron levels, that can be used to improve iron deficiency anemia in piglets.

## RESULTS

### The characterization of gut microbial community diversity in different intestinal segments of DKO weaned piglets.

**FIG 1:**
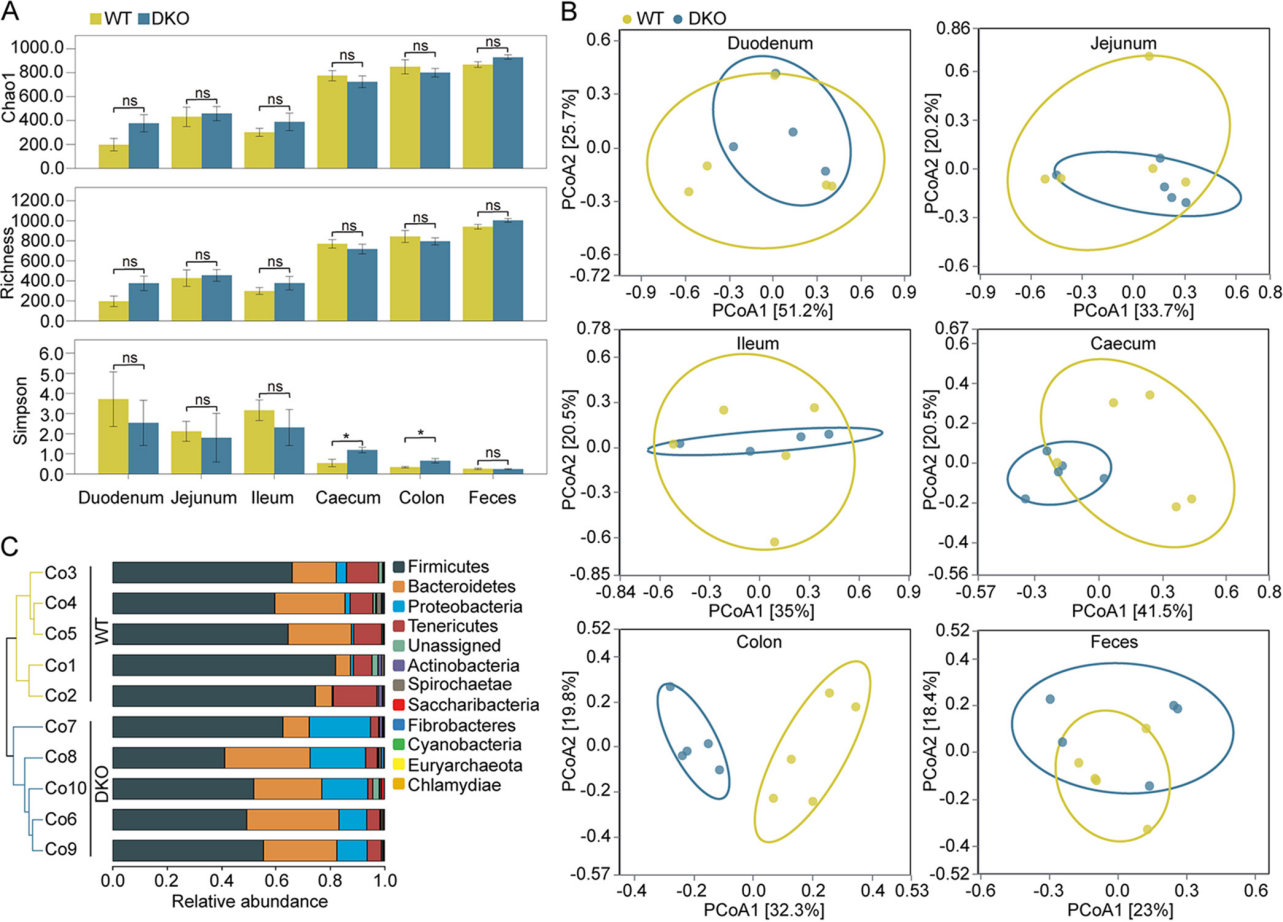
The characterization of gut microbial community diversity across different gut segments in the DKO piglets. (A) Alpha diversity indexes of the small intestinal regions (duodenum, jejunum, and ileum), large intestinal regions (cecum and colon), and feces in DKO and WT piglets. (B) Principal Coordinate Analysis (PCoA) calculated by Bray-Curtis dissimilarity between samples based on rarefied data. (C) Hierarchical clustering dendrogram of colonic microbiota in the two groups. *, *P < *0.05 (Student's *t* test). DKO group, double-gene-knockout piglets; WT group, wild-type piglets.

To characterize the gut microbiota in the weaned DKO piglets, we analyzed the microbial community in 58 samples through 16S rRNA sequencing. The samples comprised the contents of the small intestine (duodenum, jejunum, and ileum) and large intestine (cecum and colon) and feces (Fig. S1A). In the intestinal contents and feces, DKO and WT piglets displayed similar microbial diversity (measured by Chao1 and Richness indexes) (Fig. 1A). However, the DKO piglets had higher Simpson diversity indexes in the luminal regions of the cecum and colon compared to the WT piglets (*P < *0.05) (Fig. 1A). Principal coordinate analysis (PCoA) with taxonomic information did not show a clear separation of microbial community structure between DKO and WT piglets, with the exception of colonic microbiota (Fig. 1B). In the colon, a similar pattern was found by hierarchical clustering (Fig. 1C). In the WT piglets, 291 operational taxonomic units (OTUs) (22%) were shared among the small intestine samples, whereas 1,000 OTUs (35%) were shared among the large intestine samples (Fig. S1B). In the DKO piglets, 428 OTUs (31%) were shared among the small intestine samples, while 955 OTUs (34%) were shared among the large intestine samples (Fig. S1B).

### The characterization of gut microbial community composition in different intestinal segments of DKO weaned piglets.

**FIG 2:**
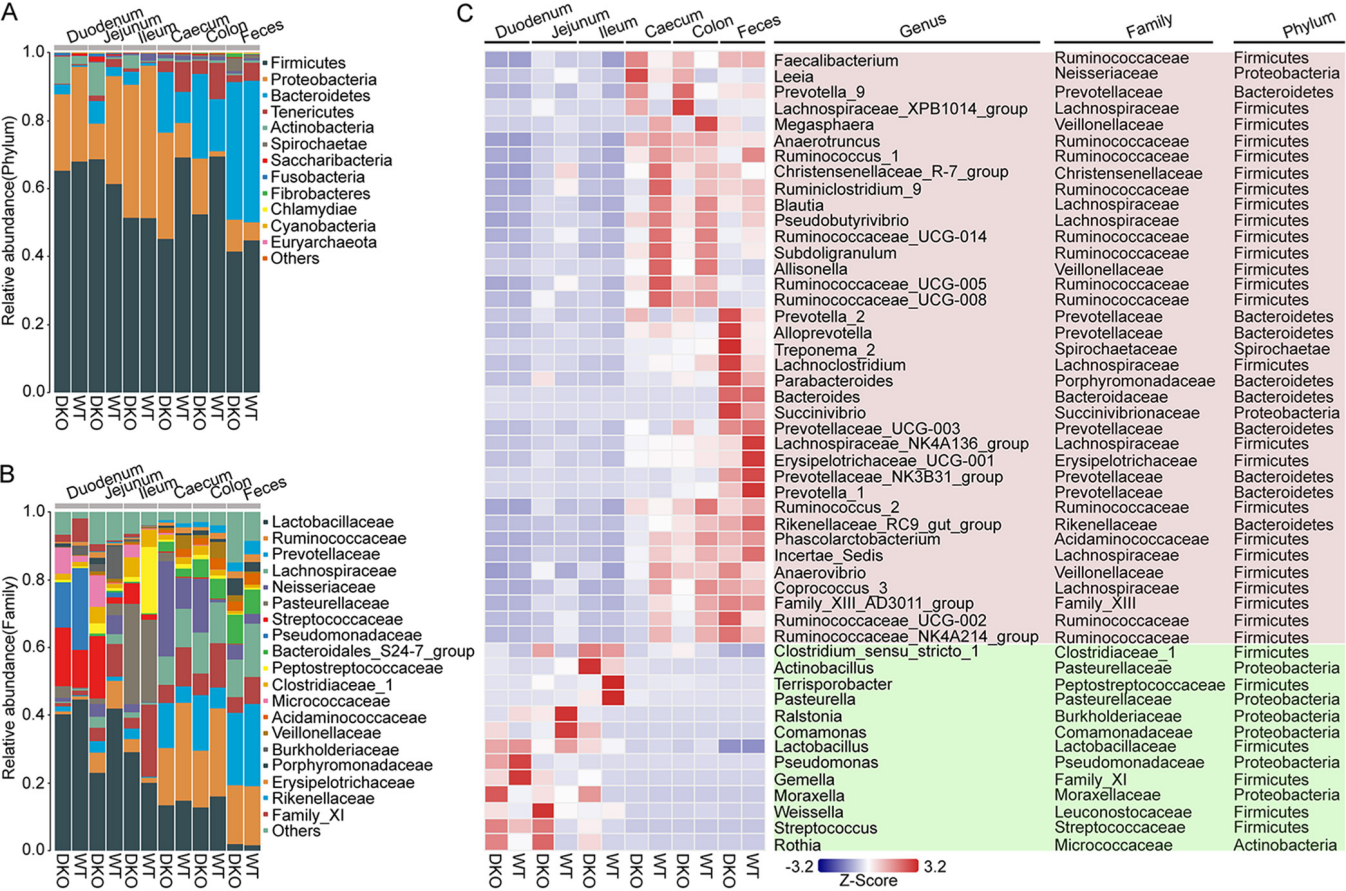
Bacterial taxonomic information in different regions of the GI tract across all samples. Relative abundances of gut microbes at the (A) phylum, (B) family, and (C) genus levels. Heatmap showing the abundance of the top 50 gut microbes at the genus level. The color of the spots in the heatmap indicates the relative abundance at the genus levels in the two groups.

Microbial community composition at the phylum, family, and genus levels was analyzed for the DKO and WT piglets. The five most abundant phyla in the small intestine were Firmicutes, Proteobacteria, Actinobacteria, Bacteroidetes, and Tenericutes, together constituting up to 97% of the OTUs in each gut segment on average (Fig. 2A). The dominating bacterial phyla in the large intestine consisted mainly of Firmicutes, Bacteroidetes, Proteobacteria, Tenericutes, and Spirochaetae (Fig. 2A). Analysis at the family level indicated that the gut microbiota was dominated by 13 major families in the small intestine: Lactobacillaceae, Pasteurellaceae, Streptococcaceae, Pseudomonadaceae, Peptostreptococcaceae, Micrococcaceae, Ruminococcaceae, Clostridiaceae_1, Burkholderiaceae, Neisseriaceae, Family_XI, Prevotellaceae, and Lachnospiraceae (Fig. 2B). However, 12 major families in the large intestine consisted mainly of Ruminococcaceae, Prevotellaceae, Lachnospiraceae, Neisseriaceae, Lactobacillaceae, Bacteroidales_S24-7_group, Acidaminococcaceae, Veillonellaceae, Rikenellaceae, Porphyromonadaceae, Erysipelotrichaceae, and Clostridiaceae_1 (Fig. 2B). The average bacterial community compositions across the GI tract at the genus level are shown in Fig. 2C. The colonization of several genera was increased in the small intestine, including *Clostridium_sensu_stricto_1*, *Actinobacillus*, *Terrisporobacter*, *Pasteurella*, *Ralstonia*, *Comamonas*, *Lactobacillus*, Pseudomonas, *Gemella*, *Moraxella*, *Weissella*, Streptococcus, and *Rothia*. However, 37 genera were enriched in the large intestinal region, such as *Faecalibacterium*, *Leeia*, *Prevotella_9*, *Lachnospiraceae_XPB1014_group*, *Megasphaera*, *Anaerotruncus*, *Ruminococcus_1*, *Christensenellaceae_R-7_group*, *Ruminiclostridium_9*, *Blautia*, *Pseudobutyrivibrio*, *Ruminococcaceae_UCG_014,* and *Subdoligranulum*.

**FIG 3:**
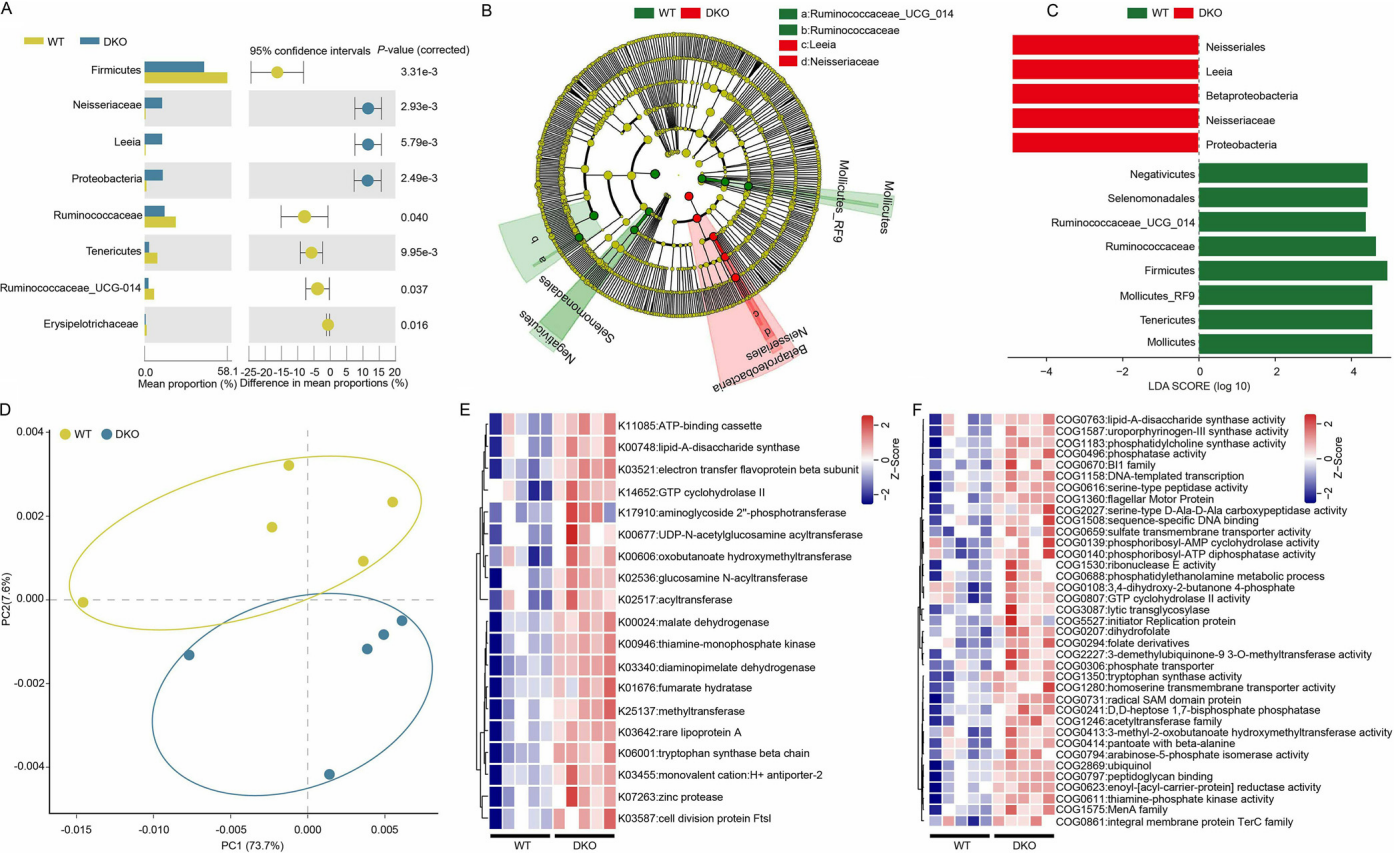
Changes in colonic microbes and function in the DKO piglets. (A) Welch’s *t* test showed that several bacterial taxa significantly differed in abundance in colonic microbiota between DKO and WT groups. The *P*-values were calculated using Welch’s *t* test with Storey’s false discovery rate comparison. (B and C) The Linear discriminant analysis (LDA) Effect Size (LEfSe) analysis of gut microbiota of DKO and WT piglets. The cladogram plot of differential species abundance in the two groups. The LDA score (≥4) plots demonstrate the taxa that were abundant in the two groups. Red indicates enriched taxa in the DKO group. Green indicates enriched taxa in the WT group. Only taxa with abundance > 0.1% of the entire sample were analyzed and shown here. (D) Principal-component analysis representation of the metagenome data sets in the colonic contents, based on abundances of functional categories obtained from KEGG functional profiles (level KO). (E) Differences in colonic microbial functions between WT and DKO groups based on KEGG functional categories (level KO) in the metagenome data sets. (F) Differences in colonic microbial functions between WT and DKO groups based on eggNOG functional profiles (level 2) in the metagenome data sets.

Welch’s *t* test was used to identify significant differences in the microbial communities at the phylum, family, and genus levels across the GI tract between DKO and WT piglets. However, the gut bacterial composition showed no obvious (*P > *0.05) differences in the feces or the luminal regions of the duodenum, jejunum, ileum, or cecum between DKO and WT piglets; the following results were for the colonic microbiota only. DKO piglets had reduced relative abundance of Firmicutes and Tenericutes and an increase in Proteobacteria compared to WT piglets (Fig. 3A). At the family level, our results showed that the relative abundance of Ruminococcaceae and Erysipelotrichaceae was significantly decreased in DKO piglets (*P < *0.05) (Fig. 3A). In contrast, Neisseriaceae was significantly enriched in DKO piglets (*P < *0.05) (Fig. 3A). At the genus level, the relative abundance of *Ruminococcaceae_UCG_014* was significantly lower in DKO piglets than in the WT piglets (*P < *0.05) (Fig. 3A). However, the DKO piglets showed remarkably increased relative abundance of *Leeia* genera (belonging to the family Neisseriaceae) compared to the WT piglets (Fig. 3A).

The linear discriminant analysis (LDA) effect size (LEfSe) method (LDA score ≥ 4) was used to identify significant colonic microbiota. The result showed that five bacterial taxa were enriched in the DKO piglets, including the Betaproteobacteria class, Neisseriales orders, Neisseriaceae families, and *Leeia* genera in the Proteobacteria phylum (Fig. 3B and C). Taxa that significantly increased in WT piglets were phylum Tenericutes (class Mollicutes and order Mollicutes_RF9), phylum Firmicutes (classes Negativicutes, orders Selenomonadales), family Ruminococcaceae and genera *Ruminococcaceae_UCG_014* (Fig. 3B and C). Analysis with the LDA score ≥ 4 further confirmed the above observation; namely, that DKO piglets showed more abundance of genera *Leeia* but less of *Ruminococca*ceae_*UCG_014*.

### Functional analysis of colonic microbiome based on metagenomic sequencing in DKO piglets.

Functional categories in the colonic microbiome between DKO and WT piglets were analyzed using metagenomic sequencing. The dominant KEGG functional categories (level 2) of colonic microbiota included genetic information processing, signaling and cellular processes, metabolism, carbohydrate metabolism, and amino acid metabolism (Fig. S1C). The principal-component analysis (PCA) showed a clear separation in KO (KEGG Orthology) between WT and DKO piglets (Fig. 3D). In addition, the functional categories were significantly differentially abundant (*P < *0.05) between WT and DKO groups (Fig. 3E). Among these categories, several functional capacities were significantly enriched in the microbiota of the DKO piglets, including K00024:malate dehydrogenase, K03340:diaminopimelate dehydrogenase, K01676:fumarate hydratase, relating to organic acid metabolism (e.g., fatty acid metabolism, fumarate metabolism, and glyoxylate and dicarboxylate metabolism) (Fig. 3F). Noteworthily, similar results were also obtained based on eggNOG analysis (Fig. 3F and Fig. S1D).

### Increased metabolites of carboxylic acids in the luminal region of the colon in DKO piglets.

**FIG 4:**
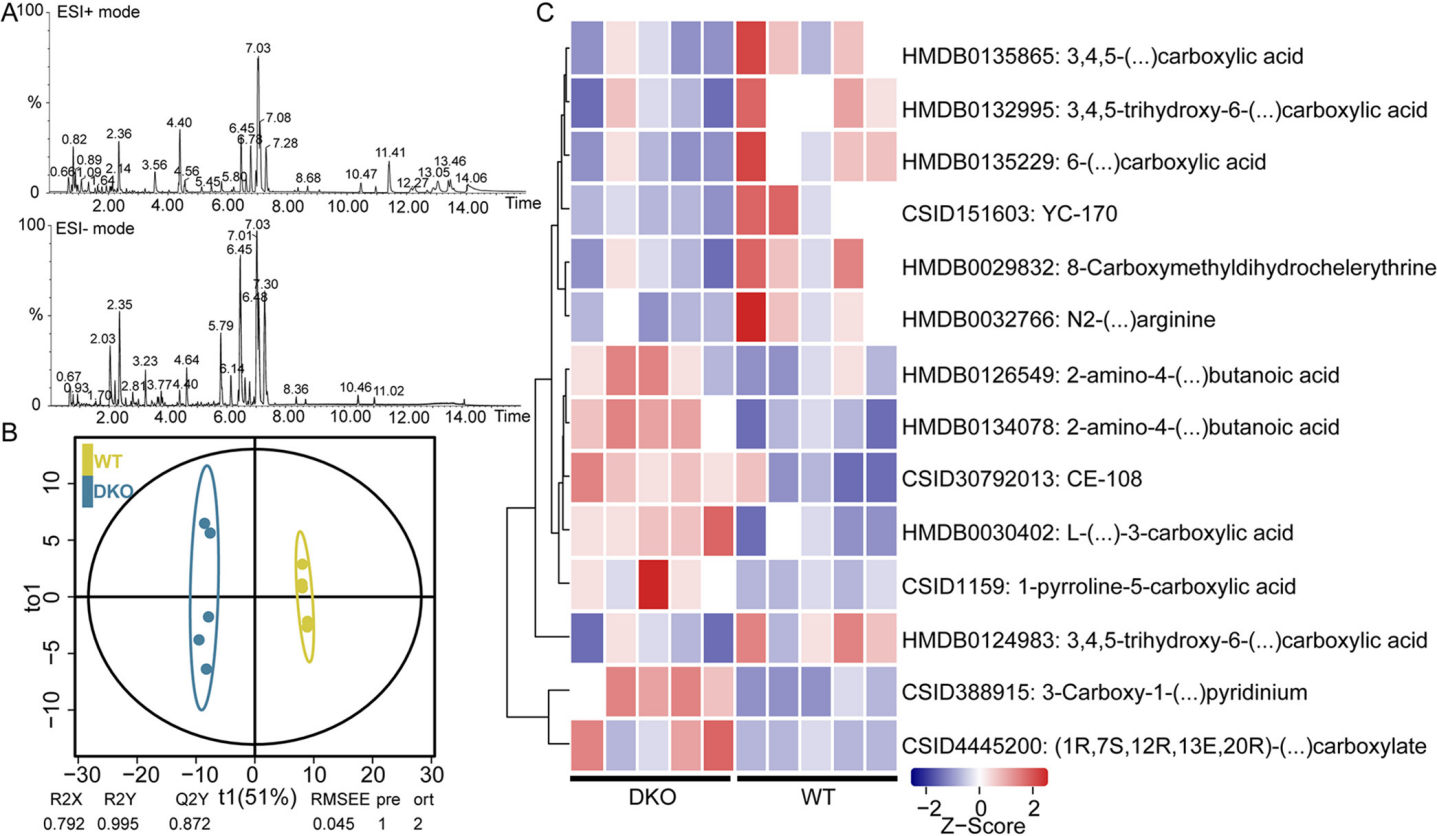
Alterations in colonic metabolites in the DKO piglets. (A) Representative LC-MS chromatograms of colon metabolomic samples. (B) The plot of orthogonal projections to latent structures discriminant analysis (OPLS-DA) for metabolites detected in ESI+ mode. (C) Comparison of the abundance of metabolites associated with the pathways of carboxylic acids and their derivatives metabolism in DKO and WT piglets.

To further explore the colonic metabolic composition, we employed a liquid chromatography–mass spectroscopy (LC-MS)-based untargeted metabolomics approach (Fig. 4A). The orthogonal projections to latent structures discriminant analysis (OPLS-DA) model showed excellent fits and was highly predictive of the piglet group from which each sample was derived (Fig. 4B; Fig. S2A). We then defined target metabolites (i.e., differentially abundant metabolites) as those with variable importance in the projection (VIP) score > 1 and *P < *0.05 in one-way analysis of variance (ANOVA). In total, 402 target metabolites were identified in DKO and WT groups, comprising 163 metabolites (ESI+ mode) and 239 metabolites (ESI-mode) (Fig. S2B and Table S1). Biomarkers from carboxylic acids metabolism were elevated in the DKO piglets and included HMDB0126549:2-amino-4-(…) butanoic acid, HMDB0134078:2-amino-4-(…) butanoic acid, CSID30792013:CE-108, HMDB0030402: L-(…)-3-carboxylic acid, CSID1159:1-pyrroline-5-carboxylic acid, HMDB0124983: 3,4,5-trihydroxy-6-(…)carboxylic acid, CSID388915: 3-Carboxy-1-(…)pyridinium, and CSID4445200:(1R,7S,12R,13E,20R)-(…)carboxylate (Fig. 4C). Additionally, the WT piglets displayed elevated levels of HMDB0135865:3,4,5-(…) carboxylic acid, HMDB0132995:3,4,5-trihydroxy-6-(…) carboxylic acid, HMDB0135229:6-(…)carboxylic acid, CSID151603:YC-170, HMDB0029832:8-Carboxymethyldihydrochelerythrine, and HMDB0032766: N2-(…)arginine (Fig. 4C).

### Increased iron levels in DKO piglets.

**FIG 5:**
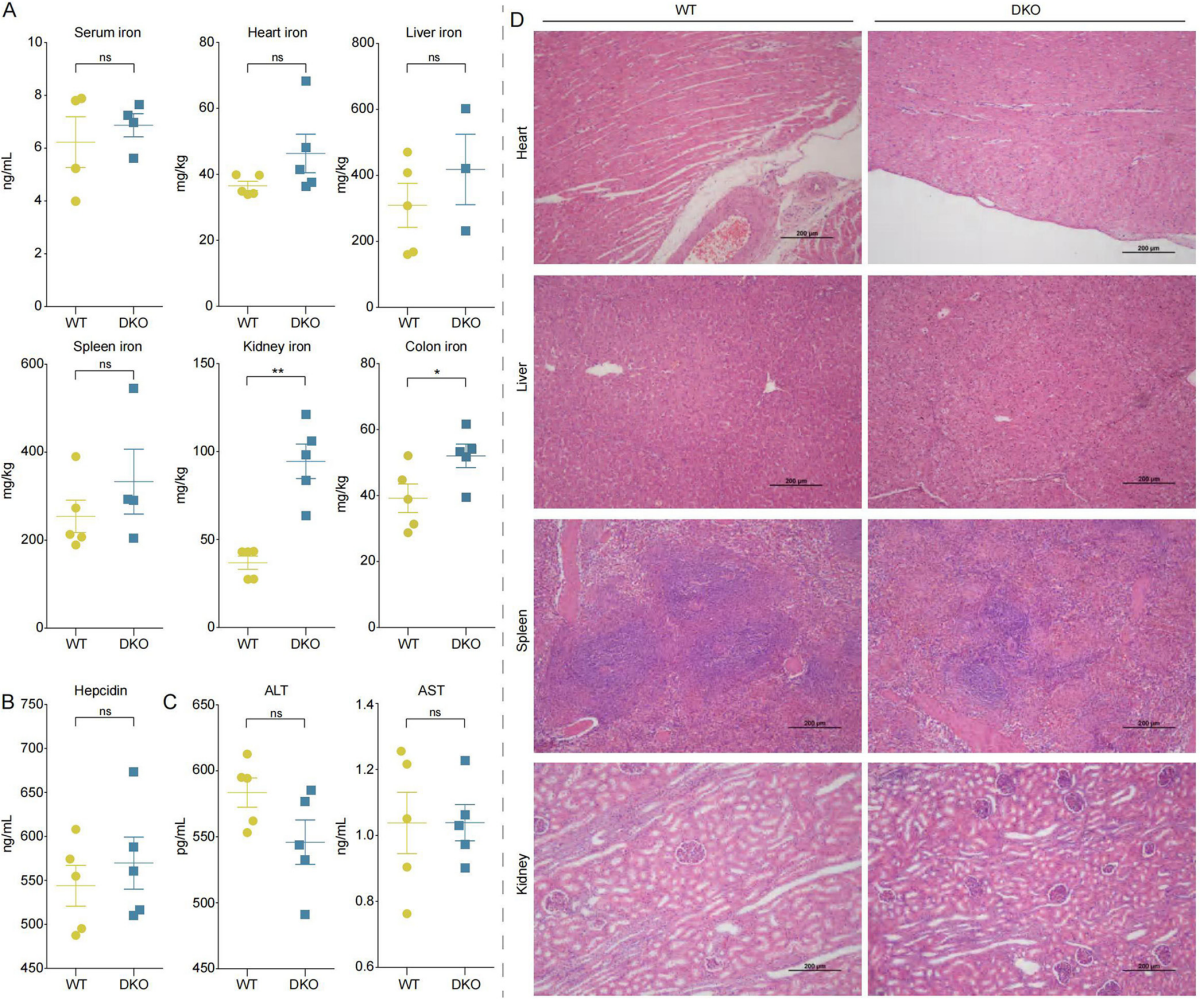
Iron status. (A) Iron levels were measured in serum and several tissues for each piglet in DKO and WT groups. (B) Hepatic hepcidin levels were measured. (C) ALT and AST activities were measured in the liver. *, *P* < 0.05; **, *P* < 0.01 (Student's *t* test). (D) Hematoxylin and eosin (H&E) staining of heart, liver, spleen, and kidney histologic sections from DKO and WT piglets. Scale bars = 200 μm.

We compared iron-related measurements of serum and tissues (i.e., heart, liver, spleen, kidney, and colon) between DKO and WT piglets without iron supplementation. The results showed mildly increased iron levels in serum, cardiac, hepatic, and splenic in DKO piglets relative to WT piglets, but the differences were not statistically significant (*P > *0.05; Student's *t* test; Fig. 5A). However, significant (kidney: *P = *0.0003; colon: *P = *0.0251; Student's *t* test) increases were found in the renal and colonic iron levels in the DKO piglets compared to the WT piglets (Fig. 5A). To reduce the likelihood of false positives, we performed nonparametric test (e.g., Mann-Whitney U Test) in the two tissues and found that the iron levels in DKO piglets were significantly (kidney: *P = *0.009; colon: *P = *0.047) increased compared to those of WT pigs. Furthermore, the mRNA expression levels were measured for iron-associated transporters and binding proteins such as *DMT1*, *TFRC*, and *FTH1* in colonic mucosa and kidney, respectively. The expression of these genes in the colonic mucosa and kidney was significantly (*P < *0.05) higher in the DKO piglets compared to WT piglets (Fig. S3A and B). As expected, Western blotting showed increased in FTH1 levels of the colonic mucosa and kidney in the DKO piglets, respectively (Fig. S3C and D). Expression of proinflammatory genes (for example *IL1B*, *TNF-α* and *CCL20*) in the colon was not significant (*P > *0.05) in the DKO piglets compared to WT piglets (Fig. S3E).

In addition, hepatic hepcidin levels did not significantly differ between DKO and WT piglets (*P > *0.05; Fig. 5B). The DKO piglets did not experience liver toxicity, as demonstrated by comparable levels to WT piglets of ALT and AST in liver tissue (*P > *0.05; Fig. 5C). Additionally, no histopathological difference in heart, liver, spleen, or kidney tissue was observed between DKO and WT piglets (Fig. 5D). These findings together demonstrated the increased iron levels in the visceral organs of the DKO weaned piglets without any pathological changes.

### Colonic microbiota and their metabolites improved host iron absorption in DKO piglets.

**FIG 6:**
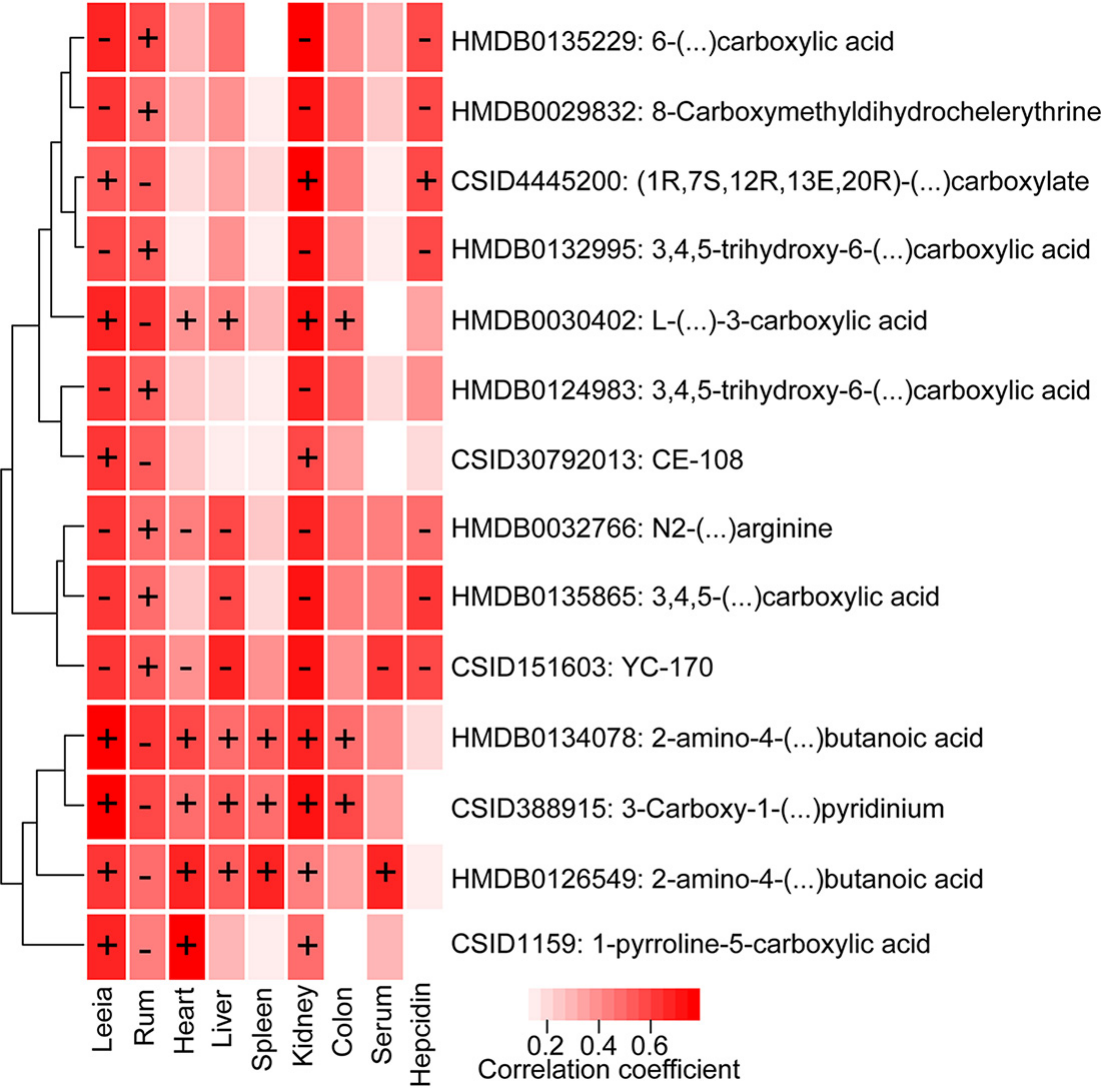
Pearson’s correlation coefficient of metabolites belonging to the “carboxylic acids and their derivatives” pathway versus *Ruminococcaceae_UCG_014* and *Leeia* genera and iron levels in serum and several tissues. Rum represents *Ruminococcaceae_UCG_014*. The heatmap represents the absolute values of Pearson's correlation coefficient. (+) indicates a positive correlation and (−) indicates a negative correlation.

To explore how colonic microbiota was associated with host iron absorption, we conducted correlation analyses between the relative abundance of gut microbiota and metabolic parameters and host iron levels. Of note, alpha diversity in the luminal region of the colon was positively correlated with iron contents in the kidney regardless of how iron was delivered systemically (Fig. S4A). We then performed Pearson's correlation analysis between iron levels and the relative abundance of gut microbiota. The correlation analysis showed positive correlations between Bacteroidetes and Proteobacteria and iron status (Fig. S4B). Inversely, the phylum Firmicutes and Actinobacteria were negatively correlated with iron status (Fig. S4B). Iron status was positively correlated with the family Neisseriaceae and Clostridiales_vadinBB60_group but negatively associated with the abundances of Ruminococcaceae, Coriobacteriaceae, and Erysipelotrichaceae (Fig. S4C). The abundances of the genera in the Ruminococcaceae family (e.g., *Ruminococcaceae_UCG_014*, *Ruminiclostridium_5*, *Ruminiclostridium_9*, *Ruminococcaceae_UCG_008,* and *Subdoligranulum*) showed the strongest negative correlation with iron levels in different tissues (Fig. S4D). In contrast, the *Leeia* genera belonging to the Neisseriaceae family and *Parabacteroides* from the family Tannerellaceae were significantly positively associated with iron status (Fig. S4D). After that, we focused on the association between target metabolites and *Ruminococcaceae_UCG_014* and *Leeia* genera and iron parameters (Fig. 6). The correlation analysis revealed that *Ruminococcaceae_UCG_014* and *Leeia* genera were highly associated with the carboxylic acids and their derivatives (Fig. 6). In addition, host iron levels were also associated with the levels of carboxylic acids and their derivatives (Fig. 6). Previous studies reported that the colonic microbiota played a critical role in host iron absorption (11, 20). Generally, approximately 15% of dietary iron is absorbed in the duodenum, and unabsorbed iron enters the colonic lumen, where it is available for microbial utilization (8). In the colon, organic acid production through microbial fermentation can lower pH values, subsequently increase iron solubility, and facilitate iron absorption (21). These significant metabolites highlighting the critical functional category, i.e., carboxylic acids metabolism regulated by the genera *Ruminococcaceae_UCG_014* and *Leeia*, probably contributed to host iron absorption.

## DISCUSSION

In this study, we investigated changes in microbial community structure across different gut segments in DKO and WT piglets. We found that colonic microbiota of DKO and WT piglets were significantly different based on using alpha- and beta-diversity analyses. At the phylum level, Firmicutes, Proteobacteria, Bacteroidetes, Tenericutes, and Actinobacteria were predominant across different gut segments, which was consistent with previous studies evaluating the microbiota in the piglets (20, 22, 23). By integrating two different analytical approaches (Welch’s *t* test and LEfSe analyses), we discovered that gene editing altered the colonic microbiota composition of weaned piglets, which was most dramatically illustrated by the reduced abundance of *Ruminococcaceae_UCG_014* genus and the increased abundance of *Leeia* genus. Furthermore, metagenomic sequence analysis revealed the enriched organic acid metabolism in the colonic microbiota of DKO piglets. Metabolome profiling further detected increases in the abundances of carboxylic acids and their derivatives in the colon contents of DKO piglets compared to WT piglets.

Iron is considered to be one of the most important trace elements for animal growth and health (13, 24). A lower iron absorption efficiency in weaned piglets compared with adult pig can result from their low digestive enzyme activity, insufficient gastric acid secretion capacity, and weak gastrointestinal motility (25, 26). Thus, iron deficiency anemia can be prevalent in piglets and usually leads to low feed intake, low growth rate, and increased chances of diarrhea (13). So far, the majority of microbiota research in piglets has been focused on how the microbiota are affected by different feeding strategies. Only very little is known about the role of the local gut microbiota in host iron absorption, which can aid in determining viable nutritional strategies to manage the intestinal health and growth development in piglets.

We found that taxa belonging to the Proteobacteria phylum (including the Neisseriaceae family and the *Leeia* genus) positively correlated with increasing host iron absorption; in contrast, members of the Erysipelotrichaceae family and *Ruminococcaceae_UCG_014* genus in the Firmicutes phylum negatively correlated with improving host iron absorption. A previous study reported that *Proteobacteria* proliferation was associated with the iron-rich environment in the GIT, which was consistent with our finding (27). The Gram-negative Neisseriaceae family was positively correlated with mediating the uptake of iron-bound proteins, including hemoglobin from the host by expressing specific receptors such as HmbRm, HpuA, and HpuB (28). However, it remains unclear whether members of the *Leeia* genus in the Neisseriaceae family is responsible for host iron absorption. A previous study showed that the Neisseriaceae family played an important role in fermenting resistant starch in the colonic segment in pigs (29). Similarly, Enterobacteriaceae, a group of Gram-negative facultative anaerobes, may be able to obtain iron over a wide range of oxygen concentrations by inducing high-affinity iron-transport systems to remove iron from host proteins (30). For example, a study observed an increase in Enterobacteriaceae in infantile iron deficiency anemia, which was different from our findings (31). The discrepancy is likely the result of differences in species or the methodologies used to identify the microbiota (20). However, the mutually advantageous relationship between these potentially pathogenic Gram-negative bacteria and host iron absorption in the gastrointestinal lumen was reported previously (30). Additionally, we found a significant negative relation between bacteria in the Firmicutes phylum and iron absorption. The Erysipelotrichaceae and Ruminococcaceae families were previously shown to be changed by iron supplementation (32). The present study suggests that these bacterial taxa in the colon have strong associations with iron absorption in piglets.

In addition to identifying iron-associated microbiome signatures, we found that gut microbial metabolites could modulate host iron absorption with untargeted metabolomics profiling. Specifically, the significantly changed colonic carboxylic acid metabolites were closely associated with *Leeia* and *Ruminococcaceae_UCG_014*, further suggesting the potential influence of *Leeia* and *Ruminococcaceae_UCG_014* on host iron absorption (8, 33). Previous studies reported that Neisseriaceae and Ruminococcaceae were involved in the metabolism of carboxylic acids (34, 35). Additionally, we found that carboxylic acids and their derivatives were associated with iron absorption. Carboxylic acids, a class of molecules that are characterized by the presence of one carboxyl group, play crucial roles in host health and nutritional metabolism (36). For example, fumaric, succinic, and citric acids could improve nonheme iron absorption by preventing ferric iron precipitation when the pH increases (37, 38). These findings indicated that colonic microbiota could effectively increase iron absorption of piglet by regulating the metabolites of carboxylic acids.

The kidney is not only regarded as a major excretory organ for elimination of metabolic wastes from the body, but also considered a major iron metabolism location (39). Gut microbiota could play a critical role in modifying renal iron absorption and homeostasis (8). Germ-free rabbits and mice showed a remarkably decreased stored iron in kidneys compared to the conventional counterparts that were colonized with a diverse and largely undefined microbiome (40, 41). However, an increased iron level in DKO kidney was found in this study, which probably was due to the modulation of colonic microbiota. It was observed that colonic tissue might play only a role in iron transport rather than in iron metabolism (42). Therefore, the kidney, but not the colon, showed the most pronounced difference in iron levels between DKO and WT piglets.

Previous studies showed that CD163 was not only an essential receptor for porcine reproductive and respiratory syndrome virus infection (43), but also played a role in promoting the clearance of plasma free hemoglobin that probably led to iron deposition in blood (44). In addition, APN, a receptor for multiple coronaviruses, is abundantly expressed on small intestinal epithelial cells. Zhang et al. discovered that pAPN might have an effect on iron transport by regulating level of transferrin receptor 1 (45). However, host iron absorption, possibly caused by genetic mutation or gut microbiota, is a complex mechanism, which needs to be further investigated.

Genome editing has been widely considered a direct, rapid, and socially acceptable method for accelerating the breeding progress to improve production traits in animals (46–48). With the development of genome-editing technology, precise modifications in livestock genome can be achieved and incorporated into selective breeding programs (48). Our laboratory previously reported that the DKO pigs were not only resistant to PRRSV and TGEV, but also maintained normal production performance (19). Despite the increased renal iron accumulation in DKO piglets, the iron levels were found within the normal range as previously reported (49). Since no pathological changes were observed in kidney for both the DKO and WT piglets, the DKO piglet would be a valuable example to obtain multiple desirable traits in a single generation.

In conclusion, we found that no significant changes in the microbial community diversity and composition in different gut segments except for colonic microbiota were detected between DKO and WT piglets. The alterations in colonic microbiota and function in the DKO piglets might play critical roles in host iron absorption. Furthermore, a panel of gut metabolites that significantly associated with iron levels was identified, including carboxylic acids and their derivatives, which might be associated with host iron absorption. Overall, our results represent an important step toward for deepening understanding of host-microbiota interactions.

## MATERIALS AND METHODS

### Experimental design and sample collections.

The experimental process for generating *CD163*/*pAPN* double knockout Large White piglets was conducted by our laboratory based as previously described (19). All piglets with the same age (DKO: *n* = 5; WT: *n* = 5) were kept in the same farrowing house (temperature 22~25°C, relative humidity 30~70%). During the lactation period, sows were fed with the basal diet without iron supplementation. Then, all piglets fed breast milk were weaned at 3 weeks and then fed the same diet formula without iron supplementation. To compare the differences in composition and function of intestinal microbiota between DKO and WT piglets, a total of 60 intestinal content specimens (*n* = 5 per group, 5 weeks of age) were collected along the entire digestive tract (including duodenum, jejunum, ileum, cecum and colon) and feces derived from the weaned piglets without iron supplementation. Due to sample contamination, we excluded one sample of the DKO piglets in the duodenum and ileum, respectively. Besides, tissues including heart, liver, spleen, kidney, and colon and blood were collected. All specimens were quickly frozen in liquid nitrogen, and then stored at −80°C until used.

### Ethics statement.

All experimental protocols were approved by the Institutional Animal Care and Use Committee (IACUC) at the Institute of Animal Sciences, Chinese Academy of Agricultural Sciences. Animal experiments were performed in strict accordance with the demands of the animal care and management of research projects (IAS2018-12).

### DNA extraction and 16S rRNA gene amplicon sequencing.

Genomic DNA of gut bacterial was extracted with a DNA extraction kit (MP bio: 116560200). DNA concentration and integrity were analyzed by measuring ratio of absorbance at 260/280 nm and agarose gel electrophoresis. Sequencing of 16S rRNA was performed as previously described (50). The V3-V4 hypervariable regions were amplified using universal primers (27F: 5′-AGAGTTTGATCCTGGCTCAG-3′; 1492R: 5′-TACGGYTACCTTGTTAYGA CTT-3′) with barcodes. The qualified libraries were sequenced on an IlluminaHiseq2500 platform followed by the standard protocol (Illumina, San Diego, CA, USA). Total bacterial copy numbers were quantified by qPCR as described by Bi et al. (51).

### 16S rRNA sequencing analysis.

We strictly controlled the quality of the 16S sequencing raw data by denoising and removing chimera with QIIME (v1.9.1, http://qiime.org/index.html). Paired-end reads were merged to tags using the mothur pipeline (52), then clustered into OTUs that were more than 97% sequence similarity. We assigned taxonomic ranks to OTU-representative sequences by searching against the Silva database using uclust within QIIME. R 4.0.5 software was used to visualizing the OTUs in different samples. The abundance of bacterial species and their adversity were calculated based on OTU and taxonomic ranks.

### Metagenomic sequencing and data processing.

Metagenomic sequencing of colonic contents (DKO group: *n* = 5; WT group: *n* = 5) was carried out according to the standard protocols as our previous description (53). Briefly, total genomic DNA was extracted from colonic contents using repeated bead-beating plus column method. Metagenomic DNA libraries were constructed using an Illumina Nextera XT kit (Illumina, San Diego, CA, USA) followed by sequencing (2 × 150 paired-end) performed on the Illumina HiSeq2500 platform at Magigene Bioinformatics Technology Co., Ltd. (Guangzhou, China).

The clean reads were obtaind by filtering adapters and low-quality reads from raw reads using Trimmomatic v0.36 (54). Then, the clean reads were aligned to the pig reference genome using SOAPdenovo (version 1.06, https://hub.nuaa.cf/aquaskyline/SOAPdenovo2), and the remaining reads were then assembled using Megahit v1.1.2 (55). Prediction of open reading frames (ORF) was conducted based on the assembled contigs by MetaGene v0.3.38 (56). The nonredundant contigs were constructed by pairwise comparison of predicted ORFs with 95% sequence identity and 90% coverage using Linclust v2.0 (57). The functional assignments of nonredundant genes were obtained based on the DIAMOND against the KEGG database release 79 (58) and eggNOG v4.5 (59), with default parameters except for -k 50 -sensitive -e 0.001. We used BLASTP version2.2.31 (http://blast.ncbi.nlm.nih.gov/Blast.cgi) to map the clean reads to the microbial reference genomes downloaded from the National Center for Biotechnology Information (http://www.ncbi.nlm.nih.gov). The taxonomic relative abundance profile was calculated using HUMAnN v2.0 (https://huttenhower.sph.harvard.edu/humann2/).

### Metabolomics.

The colonic contents for UPLC-QTOF-MS were prepared as described in a previous study (60). In short, each sample was mixed with 300 mL chromatographic acetonitrile followed by vortexing. After centrifugation at 12,000 × *g* at 4°C for 15 min, the supernatant was analyzed with untargeted LC-MS.

The clean data were processed with orthogonal partial least-squares-discriminant analysis (OPLS-DA) in SIMCA-P 14.0 software (Umetrics AB, Umea, Sweden), and the resulting score plots were used to visualize the model. Molecular structures of interest were defined as those with variable importance in the projection (VIP) >1 and *P < *0.05 (*t* test for filtered data).

### Iron-related measurements.

Nonheme iron levels in the heart, liver, spleen, kidney, and colon were determined by a wet ashing procedure as previously described (61). Serum iron, liver hepcidin, alanine aminotransferase (ALT), and aspartate aminotransferase (AST) levels were also measured using an enzyme-linked immunosorbent assay (ELISA) kit (Cat.number ml836594-J, Porcine FE ELISA KIT) following the manufacturer’s instructions.

### Histology.

During the necropsy, piglet heart, liver, spleen, and kidney tissues were collected. The tissues were fixed overnight according to the previously described (19). Subsequently, the sections were stained with hematoxylin and eosin, and images were captured using an Infinity HD Lumenera digital camera.

### Quantitative real-time PCR (qPCR) and Western blotting analyses.

The colonic mucosa and renal tissues were collected from WT and DKO piglets. The qPCR and Western blotting analyses were performed as detailed in a previous study (19). The 18S rRNA gene was used as the internal control for target mRNA levels normalization. Primer sequences are shown in Table S2. The antibodies for Western blotting were anti-Ferritin Heavy Chain (ab231253: Abcam, Cambridge, UK), GAPDH (14C10) Rabbit MAb (2118S, Cell Signaling Technology, Danvers, MA, USA) and anti-rabbit IgG HRP-linked antibody (7074S, Cell Signaling Technology, Danvers, MA, USA).

### Statistical analysis.

All data were presented as the means ± standard error of the mean (SEM), with the exception of microbial community data. The Student's *t* test and Mann-Whitney U test were used for assessing the significant difference between groups. For linear discriminant analysis effect size (LEfSe) analysis, a *P* value < 0.05 for the Kruskal–Wallis test and the linear discriminant analysis (LDA) score ≥ 4 were used to define the significance. Additionally, differential microbiota was defined as those with a corrected *P < *0.05 with the Welch’s *t* test correction. Pearson's correlation coefficient was calculated in SPSS Statistics 22 (SPSS Inc., Chicago, IL, USA). Heatmaps were generated using the ggplot2 package (https://www.rdocumentation.org/packages/ggplot2/versions/2.1.0) in R.

### Data availability.

The raw sequencing data generated in this study has been deposited in the NCBI SRA database with the accession numbers PRJNA893407 (16S rRNA sequencing) and PRJNA894240 (metagenomic sequencing).
