# Supplementary material for: Characterization of Gut Microbiota Compositions along the Intestinal Tract in CD163/pAPN Double Knockout Piglets and Their Potential Roles in Iron Absorption
Source: Microbiol Spectr. 2023 Jan 10;11(1):e01906-22. doi: 10.1128/spectrum.01906-22 (PMC9927099; doi:10.1128/spectrum.01906-22)
Supplement: Supplemental file 1 — Fig. S1-S4. Download spectrum.01906-22-s0001.pdf, PDF file, 0.6 MB [file spectrum.01906-22-s0001.pdf]

Characterization of gut microbiota compositions along the intestinal tract in *CD163/pAPN* double knockout piglet and their potential roles on iron absorption

Xiu-Ling Zhang[a,b], Yan-Rong Zhou[c], Song-Song Xu[d], Si Xu[c], Yu-Jian Xiong[c], Kui Xu[a], Chang-Jiang Xu[a], Jing-Jing Che[a], Lei Huang[d], Zhi-Guo Liu[a], Bing-Yuan Wang[a], Yu-Lian Mu[a], Shao-Bo Xiao[c], Kui Li[a,d]

[a] State Key Laboratory of Animal Nutrition, Institute of Animal Sciences, Chinese Academy of Agricultural Sciences, Beijing 100193, China.

[b] College of Animal Science and Technology, Nanjing Agricultural University, Nanjing 210095, China.

[c] State Key Laboratory of Agricultural Microbiology and Key Laboratory of Preventive Veterinary Medicine in Hubei Province, College of Veterinary Medicine, Huazhong Agricultural University, Wuhan 430070, China.

[d] Shenzhen Branch, Guangdong Laboratory of Lingnan Modern Agriculture, Genome Analysis Laboratory of the Ministry of Agriculture and Rural Affairs, Agricultural Genomics Institute at Shenzhen, Chinese Academy of Agricultural Sciences, Shenzhen 518120, China.

Xiu-Ling Zhang, Yan-Rong Zhou and Song-Song Xu contributed equally to this work.

Corresponding authors: Kui Li, likui@caas.cn; Shaobo Xiao, vet@mail.hzau.edu.cn; Yulian Mu, mouyulian@caas.cn;

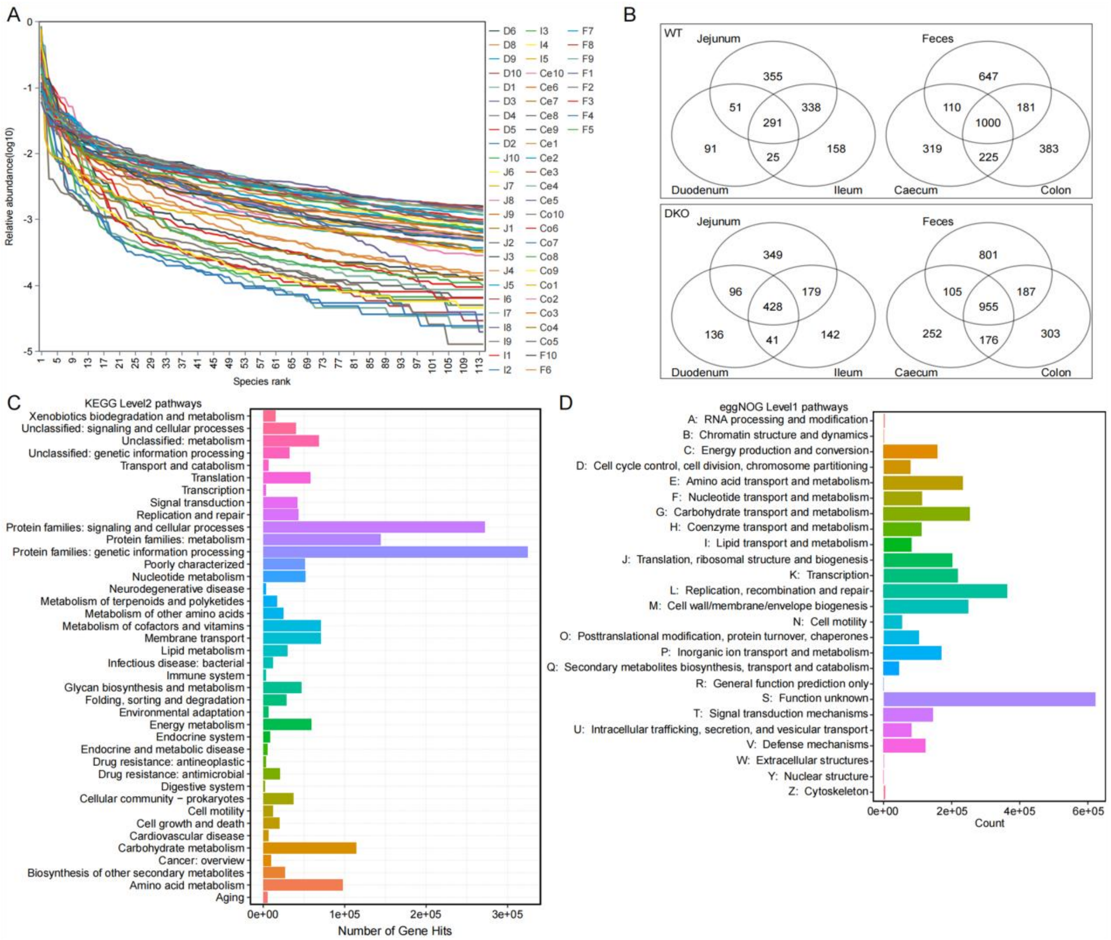

FIG S1 (A) Rank abundance curves indicate that the richness and evenness of microbial species in DKO group are comparable to those of the WT group. (B) Venn diagrams showing shared OTUs in the small and large intestine, respectively. (C) Functional composition of colonic microbiota at level 2 KEGG functional categories in the metagenome datasets. (D) Functional composition of colonic microbiota at level 1 eggNOG functional categories in the metagenome datasets.

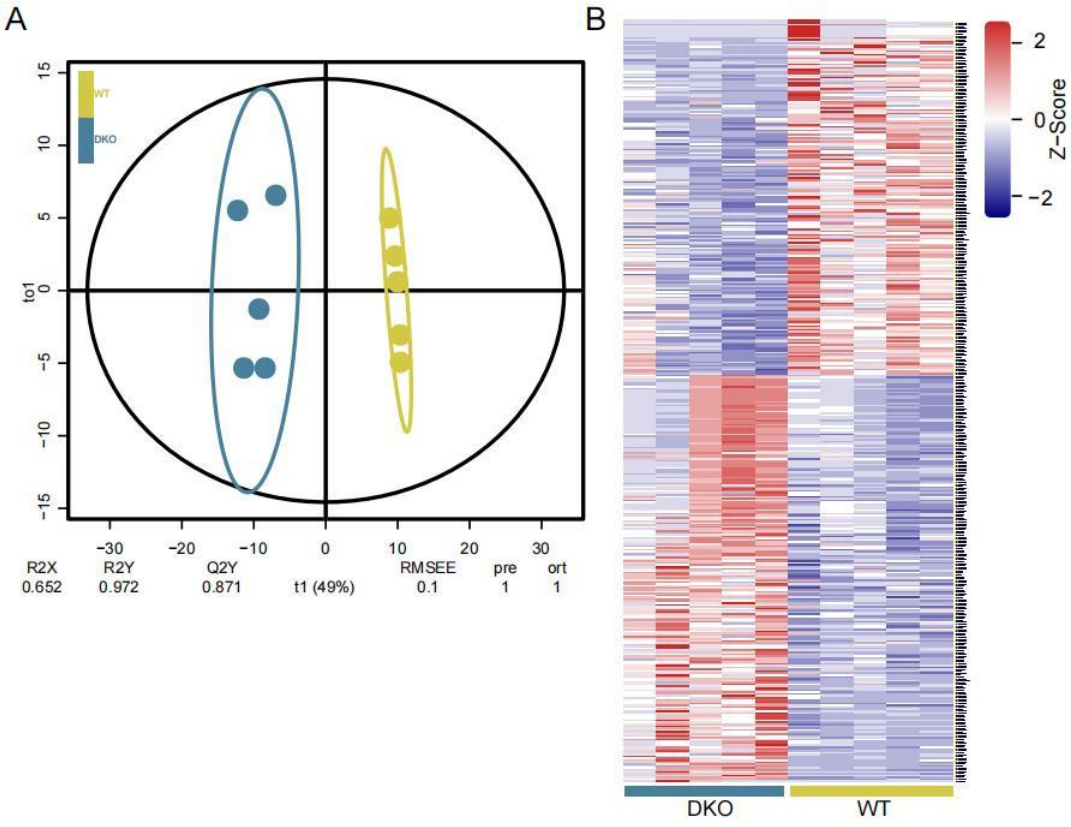

FIG S2 (A) OPLS-DA plot based on the metabolites detected in negative electrospray ionization (ESI-) mode. (B) Heatmaps depicting significant annotated metabolites with VIP > 1 and unpaired *t*-test $p < 0.05$ between DKO and WT groups.

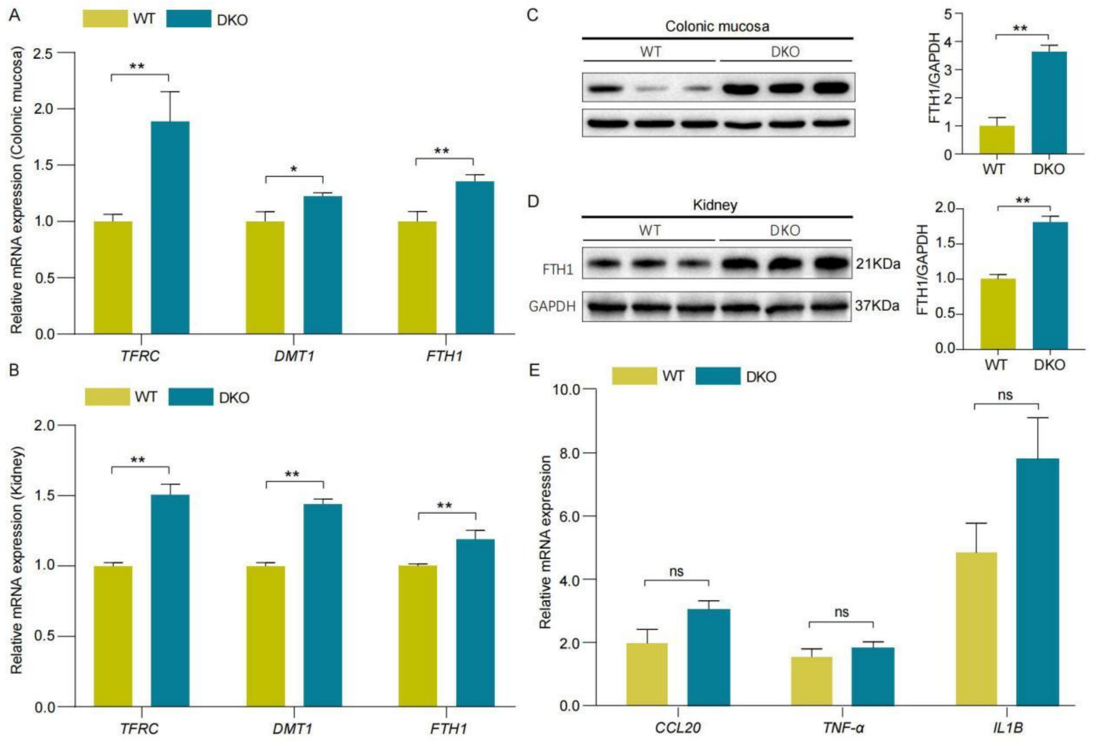

Fig S3 (A) qPCR analyses of colonic mucosa iron transporters and binding proteins in DKO and WT piglets. (B) qPCR analyses of renal iron transporters and binding proteins in DKO and WT piglets. (C) Colonic mucosa protein expression of FTH1 in DKO and WT piglets. (D) Renal protein expression of FTH1 in DKO and WT piglets. (E) qPCR analyses of pro-inflammatory molecules in the colon. Data are shown as the mean ± SEM. **P* < 0.05; ***P* < 0.01 (student's *t*-test).

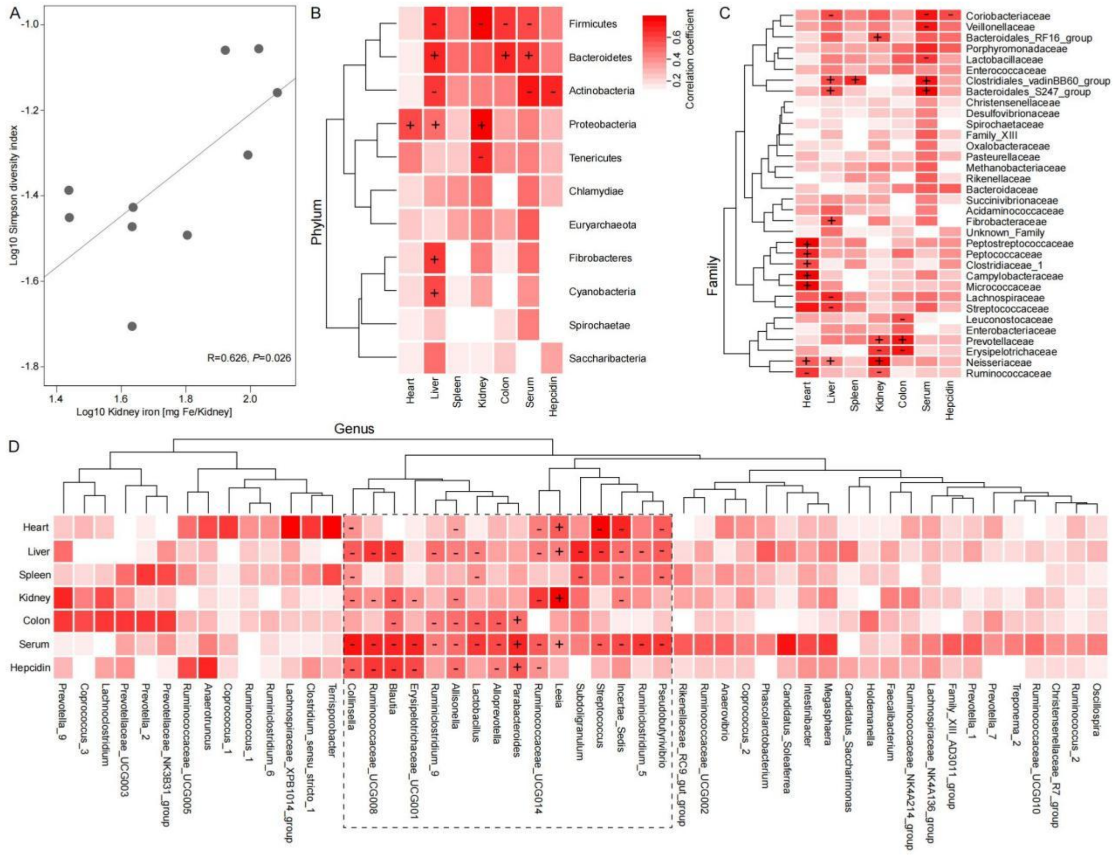

Fig S4 (A) The association Simpson diversity index in colonic microbiota and renal iron for each piglet in DKO and WT groups (both axes displayed as $\log_{10}$). Pearson's correlation coefficient is shown. (B, C and D) Correlation of colonic bacterial taxa with iron status. Heatmap of Pearson's correlation between iron levels and bacterial at the phylum, family, and genus levels for all piglets combined from DKO and WT groups. (+) indicates a positive correlation and (-) indicates a negative correlation. The dashed box represents the gut microbiota at the genus level were associated with iron levels.
